# AEBP1 is a Novel Oncogene: Mechanisms of Action and Signaling Pathways

**DOI:** 10.1155/2020/8097872

**Published:** 2020-05-27

**Authors:** Amin F. Majdalawieh, Mariam Massri, Hyo-Sung Ro

**Affiliations:** ^1^Department of Biology, Chemistry, and Environmental Sciences, College of Arts and Sciences, American University of Sharjah, Sharjah, UAE; ^2^Department of Biochemistry and Molecular Biology, Faculty of Medicine, Dalhousie University, Halifax, NS, Canada

## Abstract

Adipocyte enhancer-binding protein 1 (AEBP1) is a transcriptional repressor involved in the regulation of critical biological processes including adipogenesis, mammary gland development, inflammation, macrophage cholesterol homeostasis, and atherogenesis. Several years ago, we first reported the ability of AEBP1 to exert a positive control over the canonical NF-*κ*B pathway. Indeed, AEBP1 positively regulates NF-*κ*B activity via its direct interaction with I*κ*B*α*, a key NF-*κ*B inhibitor. AEBP1 overexpression results in uncontrollable activation of NF-*κ*B, which may have severe pathogenic outcomes. Recently, the regulatory relationship between AEBP1 and NF-*κ*B pathway has been of great interest to many researchers primarily due to the implication of NF-*κ*B signaling in critical cellular processes such as inflammation and cancer. Since constitutive activation of NF-*κ*B is widely implicated in carcinogenesis, AEBP1 overexpression is associated with tumor development and progression. Recent studies sought to explore the effects of the overexpression of AEBP1, as a potential oncogene, in different types of cancer. In this review, we analyze the effects of AEBP1 overexpression in a variety of malignancies (e.g., breast cancer, glioblastoma, bladder cancer, gastric cancer, colorectal cancer, ovarian cancer, and skin cancer), with a specific focus on the AEBP1-mediated control over the canonical NF-*κ*B pathway. We also underscore the ability of AEBP1 to regulate crucial cancer-related events like cell proliferation and apoptosis in light of other key pathways (e.g., PI3K-Akt, sonic hedgehog (Shh), p53, parthanatos (PARP-1), and PTEN). Identifying AEBP1 as a potential biomarker for cancer prognosis may lead to a novel therapeutic target for the prevention and/or treatment of various types of cancer.

## 1. Introduction

**Figure 1:**
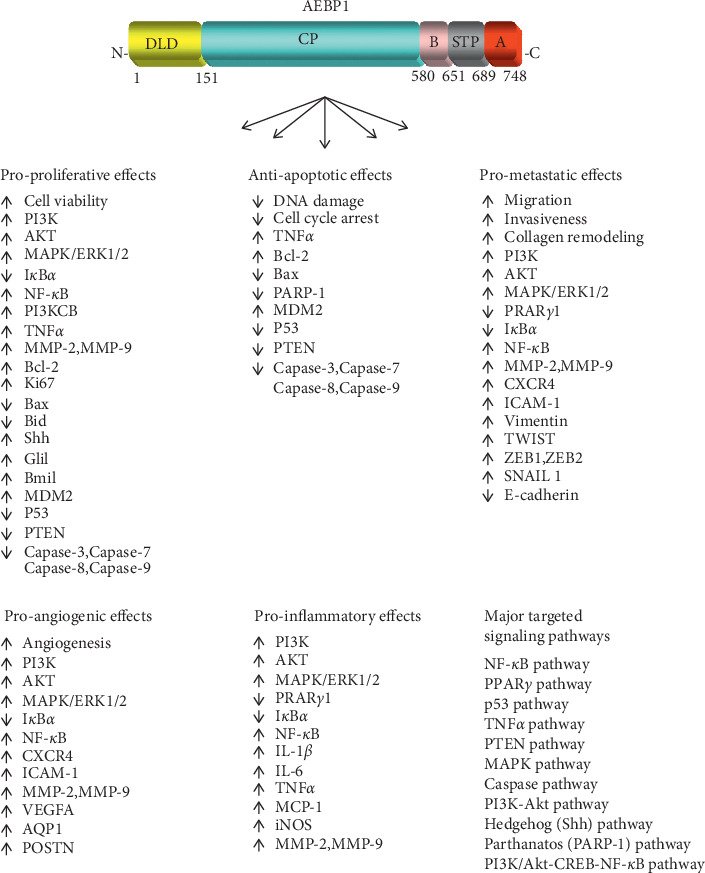
An overview of the main regulatory effects of AEBP1 on cancer-related proteins and the major signaling pathways targeted by AEBP1 to promote its proproliferative, antiapoptotic, prometastatic, proangiogenic, and proinflammatory effects, triggering cancer development. AEBP1 protein possesses two well-characterized domains, a discoidin-like domain (DLD) at its N-terminus and a central carboxypeptidase (CP) domain. The C-terminus of AEBP1 contains three subdomains: a basic (B) subdomain, a serine-threonine-proline rich (STP) subdomain, and an acidic (A) subdomain.

Adipocyte enhancer-binding protein 1 (AEBP1) is a ubiquitously expressed, multifunctional protein, with high levels of expression in preadipocytes and macrophages [1]. AEBP1 was initially characterized as a transcriptional repressor of the adipose P2 (aP2) gene in preadipocytes, and its expression is downregulated during adipocyte differentiation [2, 3]. Aortic carboxypeptidase-like protein (ACLP) is an N-terminally extended, nonnuclear isoform of AEBP1, which is upregulated during vascular smooth muscle cell differentiation [4]. AEBP1 gene gives rise to AEBP1 and ACLP mRNAs by alternative splicing, suggesting a novel example of the regulation of subcellular localization by protein truncation [1]. AEBP1 consists of an N-terminal discoidin-like domain (DLD), a carboxypeptidase (CP) domain, and a C-terminal DNA-binding domain (DBD) (Figure 1). AEBP1 is found equally distributed between the nucleus and cytosol [5] and can alter signaling through protein-protein interaction in the cytosol and by transcriptionally repressing anti-inflammatory and apoptotic genes in the nucleus [6–11].

AEBP1 exerts its regulatory functions in a myriad of ways. AEBP1 can regulate gene expression or exert transcriptional suppression by binding to specific DNA-binding sites of targeted genes. For example, AEBP1 was demonstrated to play a critical role in adipocyte differentiation by negatively regulating the adipose P2 (aP2) gene in preadipocytes [2]. AEBP1 binds to the adipocyte enhancer-1 (AE-1) DNA sequence on the aP2 gene, leading to the transcriptional repression of aP2 [2]. AEBP1 expression significantly decreases in mature adipocytes, compared to preadipocytes, strongly indicating that AEBP1 plays a negative regulatory role in adipocyte differentiation [2, 6]. Studies have also illustrated the ability of AEBP1 to induce transcriptional regulation of macrophage cholesterol homeostasis [8, 12]. AEBP1 can transcriptionally suppress the activity of PPAR*γ*1 and LXR*α* in macrophages, which are nuclear receptors that transactivate proteins involved in cholesterol efflux and reverse cholesterol transport [8, 13]. The promoter regions of PPAR*γ*1 and LXR*α* possess a homologous sequence to AE-1, previously shown to be an AEBP1-binding sequence [13]. AEBP1 downregulates the activity of PPAR*γ*1 and LXR*α* by binding to the AE-1 homologous sequence, consequently suppressing the downstream target genes ABCA1, ABCG1, and ApoE and consequently inhibiting cholesterol efflux [13]. The transcriptional suppression of PPAR*γ*1 and LXR*α* renders AEBP1 as a critical modulator of macrophage cholesterol homeostasis.

AEBP1 has also been shown to regulate the transcriptional activation of molecules within a signaling pathway, thereby influencing the activity of a specific biological process. For instance, AEBP1 can transcriptionally suppress adipocyte differentiation by regulating the activation of MAPK/ERK pathway [6]. It was reported that MAPK-mediated phosphorylation of PPAR*γ*1 results in downregulation of PPAR*γ*1 activity and the subsequent inhibition of adipose differentiation [14]. In line with these findings, we have previously demonstrated that AEBP1 directly interacts with ERK1/2, leading to MAPK activation and subsequent suppression of adipocyte differentiation [6]. These results highlight AEBP1 and MAPK as key regulators of adipogenesis.

NF-*κ*B pathway is a complex signaling mechanism involved in the regulation of vital processes including DNA transcription, cell survival, and inflammatory responses [15, 16]. NF-*κ*B is a family of transcription factors that are ubiquitously expressed in nearly all cell types. NF-*κ*B proteins include NF-*κ*B/p50, NF-*κ*B/p52, NF-*κ*B/p65 (ReIA), ReIB, and c-Rel [16]. These proteins have a structural similarity in that they share a Rel homology domain (RHD) at the N-terminus [17]. In the cytosol, NF-*κ*B is found in its inactive form and is activated by various stimuli [18, 19]. The activation of NF-*κ*B is regulated by NF-*κ*B inhibitors, namely, I*κ*B*α* and I*κ*B*β* [20]. I*κ*B*α* and I*κ*B*β* are characterized by ankyrin (ANK) repeats that interact with the RHD domains on NF-*κ*B proteins, thereby inactivating NF-*κ*B [17]. Moreover, the promoter region of I*κ*B protein possesses a *κ*B DNA-binding site, facilitating the upregulation of I*κ*B proteins upon NF-*κ*B activation. The ability of NF-*κ*B to upregulate I*κ*B*α* and I*κ*B*β* ensures a precise and balanced regulation of NF-*κ*B activation [21]. The NF-*κ*B signaling module consists of two pathways: canonical (also known as classical) and noncanonical (also known as alternative) [19]. The formation and activation of the IKK complex, which consists of catalytically active kinases (e.g., IKK*α*, IKK*β*, and IKK*γ*) and noncatalytic regulatory proteins (e.g., NEMO and ELKS), are universal events in both signaling pathways. In the canonical signaling pathway, ligand binding to a cell surface receptor leads to the recruitment of adaptor proteins (e.g., TRAF6) to the receptor, leading to the recruitment of IKK complex and subsequent phosphorylation and degradation of the I*κ*B proteins. Unlike the canonical signaling pathway, the noncanonical signaling pathway, which is normally triggered by nonproinflammatory cytokines (e.g., LT*β*, BAFF, and CD40L) as well as some viruses (e.g., HTLV and EBV), does not allow the recruitment of NEMO. Instead, ligand binding to a cell surface receptor leads to the recruitment of NIK, which in turn phosphorylates and activates IKK*α* homodimers, leading to transactivation of a signal that culminates in NF-*κ*B activation. Typically, the canonical signaling pathway leads to the activation of NF-*κ*B dimers consisting of NF-*κ*B/p50, NF-*κ*B/p65, RelB, and c-Rel, while the noncanonical signaling pathway leads to the activation of NF-*κ*B dimers consisting primarily of NF-*κ*B/p52 and RelB [19].

AEBP1 is highly expressed in macrophages and was shown to stimulate the expression of several inflammatory mediators including interleukin-6 (IL-6), tumor necrosis factor *α* (TNF*α*), monocyte chemoattractant protein 1 (MCP-1), and inducible NO synthase (iNOS) [8]. A follow-up study revealed that AEBP1 expression in macrophages enhances inflammatory responses via upregulation of NF-*κ*B activation [9]. AEBP1 interacts with I*κ*B*α* in macrophages via DLD, inducing the phosphorylation and proteolytic degradation of I*κ*B*α* and promoting NF-*κ*B/p65 activity [9]. However, the specific region of I*κ*B*α* that mediates the interaction with DLD of AEBP1 is yet to be identified. I*κ*B*α* is known to translocate to the nucleus upon synthetization to bind to NF-*κ*B, and about 50% of AEBP1 protein population is sequestered in the nucleus [9]. Thus, it is possible that AEBP1 interacts with I*κ*B*α* in the nucleus, leading to the upregulation of NF-*κ*B/p65 transcriptional activity [19].

Modulation of AEBP1 expression has been implicated in various biological processes and several chronic diseases. For example, abnormal upregulation of AEBP1 expression was associated with Alzheimer's disease (AD) [22–24]. AEBP1 overexpression was associated with the progression of brain atrophy in AD [23] and the progression of amyloid *β* plaques [24]. Interestingly, the nuclear localization of NF-*κ*B/p65 was observed in specific neurons with AEBP1 overexpression [24]. AEBP1 has also been shown to play a role in abdominal aortic aneurysm (AAA) [25]. AEBP1 overexpression in AAA results in the activation of NF-*κ*B/p65 and the consequent upregulation of inflammatory cytokines IL-1*β*, IL-6, TNF*α*, and MCP-1, as well as MMP2 and MMP-9, matrix metalloproteinases (MMPs) [25]. In addition, modulation of AEBP1 expression has been implicated in other processes and conditions such as adipogenesis [2, 5, 6, 11, 26, 27], mammary gland development [28–30], macrophage cholesterol homeostasis [8, 12, 31], inflammation [8, 25, 28, 32], and atherosclerosis [32].

The role of AEBP1 in promoting carcinogenesis was recently investigated by several research groups. Given that AEBP1 overexpression is implicated in chronic and inflammatory diseases, recent studies have sought to analyze the characteristics of AEBP1 in different forms of cancer. The upregulation of AEBP1 mRNA and protein expression was reported in various cancer samples, supporting the role of AEBP1 in cancer progression. AEBP1 was shown to be methylated in several renal cell carcinoma cell lines and tumor samples [33]. Moreover, mutations that lead to the constitutive stimulation of NF-*κ*B signaling have been strongly associated with the development and progression of cancer [34]. NF-*κ*B proteins are considered as important oncogenes that augment a number of metastatic features including cell growth, cell proliferation, and inflammation [35, 36]. Since AEBP1 mediates the activation of NF-*κ*B signaling, and since the uncontrollable activation of NF-*κ*B promotes oncogenesis, it is suggested that AEBP1 overexpression may promote tumor progression via upregulation of NF-*κ*B pathway. Herein, we analyze the mechanisms by which AEBP1 promotes tumorigenesis and the key signaling pathways implicated in this regulation, focusing on the positive relationship between AEBP1 and NF-*κ*B pathway. AEBP1 has also been observed to contribute to metastasis and cancer development by modulating various other signaling pathways including PI3K-Akt, sonic hedgehog (Shh), p53 (a tumor suppressor), parthanatos (PARP-1), and PTEN, all of which will be comprehensively discussed in this review. Most importantly, investigating the direct association between AEBP1 and tumorigenesis can yield significant implications as AEBP1 may prove to be an effective biomarker for cancer prognosis and a therapeutic target for prevention and/or treatment of cancer.

## 2. AEBP1 Promotes Tumorigenesis via Canonical NF-*κ*B Signaling Pathway

**Figure 2:**
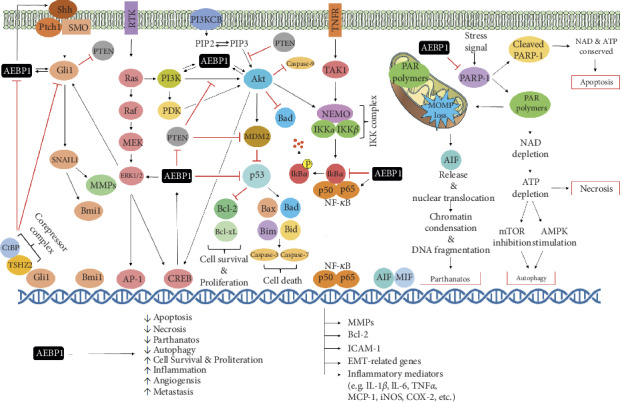
A schematic, mechanistic representation of the molecular networks and signaling pathways that underlie the molecular roles of AEBP1 in carcinogenesis.

NF-*κ*B signaling pathway is known to regulate a number of biological functions including cell proliferation, apoptosis, differentiation, and inflammation [37]. Dysregulation or hyperactivation of NF-*κ*B pathway was shown to promote the development and metastasis in many human cancers [38]. AEBP1 has been reported to play a role in the tumorigenesis of different types of cancers via abnormal activation of NF-*κ*B pathway (Table 1). The main regulatory effects of AEBP1 on cancer-related proteins involved in canonical NF-*κ*B pathway are illustrated in Figure 1.  Figure 2 depicts the positive relationship between AEBP1 and canonical NF-*κ*B signaling.

### 2.1. Breast Cancer

AEBP1 is implicated in mammary gland development and tumorigenesis through its interference with the mammary epithelial-stromal microenvironment [28–30]. Holloway and colleagues [28] demonstrated that AEBP1 overexpression in stromal macrophages triggers mammary epithelial cell hyperplasia. Transgenic mice, with targeted AEBP1 overexpression (AEBP1^TG^) in adipocytes and macrophages, were used to study the role of AEBP1 in mammary tumorigenesis. Alveolar hyperplasia developed in 30% of 30-week old AEBP1^TG^ mice, compared to nontransgenic mice (AEBP1^NT^). Interestingly, 100% of 30-week-old AEBP1^TG^ mice exhibited alveolar hyperplasia when subjected to a high-fat diet (HFD). Moreover, immunohistochemical analysis revealed a high rate of stromal macrophage infiltration in mammary epithelial cells of AEBP1^TG^ mice, which was further augmented upon HFD administration. These results indicate that overexpression of stromal-derived AEBP1, along with an HFD, instigates mammary tumorigenesis *in vivo.*

Importantly, AEBP1 was shown to promote proinflammatory signaling in mammary glands through the upregulation of NF-*κ*B/p65 in stromal macrophages [28]. Mammary glands isolated from AEBP1^TG^ mice expressed about 4-fold higher concentration of nuclear NF-*κ*B/p65 compared to those isolated from AEBP1^NT^ mice. A ^32^P-labeled kB DNA-binding consensus sequence revealed that the transcriptional activity of nuclear NF-*κ*B/p65 correlated with AEBP1 overexpression, indicating that mammary tumorigenesis is promoted by AEBP1-mediated activation of NF-*κ*B pathway. Another study by Zhou and colleagues [30] identified AEBP1 as one of the transcriptional factors that regulates the normal development of mammary glands. Specifically, AEBP1 was shown to be involved in the involution process as it promotes macrophage inflammatory response during apoptosis [30]. Since AEBP1 is shown to induce inflammatory responses during mammogenesis, it supports the observation that AEBP1 overexpression leads to mammary gland hyperplasia. Furthermore, augmented expression of TNF*α* was detected in mammary glands of AEBP1^TG^ mice. A bone marrow transplantation experiment coupled with *γ*-irradiation established that the source of TNF*α* was stromal macrophages, strongly indicating that stromal AEBP1 promotes NF-*κ*B-mediated proinflammatory responses via TNF*α* signaling, leading to mammary tumorigenesis [28].

Another study, using massively parallel signature sequencing (MPSS), identified AEBP1 as one of several differentially expressed genes in malignant breast epithelial cells [39]. Analysis of the total RNA showed that AEBP1 transcript was overexpressed in malignant breast epithelium. A gene set enrichment analysis (GSEA) reported that AEBP1 was a highly enriched myoepithelium-type gene in malignant breast tumors. Furthermore, the biological function of AEBP1 was significantly associated with the skeletal development gene subset (GO:0001501) according to a gene ontology analysis. Patients with metastatic breast cancer are known to experience considerably weak skeletal complications [55]. Interestingly, AEBP1 plays a role in the molecular pathway of bone osteoblastic module, a module that has been implicated in the progression of several tumors including breast cancer [56]. Specifically, the role of AEBP1 in the bone osteoblastic module was associated with differentiation and matrix remodeling of osteoblasts [56]. Thus, AEBP1 overexpression may be a prominent factor in tumor progression of malignant breast cancer cells through bone differentiation and matrix remodeling. NF-*κ*B pathway is reported to play a role in the differentiation and remodeling of bone cells and was further shown to promote tumor-mediated osteolysis in breast cancer [57–59]. In line with these observations, we speculate that AEBP1 elicits the development and metastasis of malignant breast cancer cells via the canonical NF-*κ*B pathway. Collectively, these studies highlight AEBP1 as a potential therapeutic target for the inhibition of metastatic breast cancer.

### 2.2. Glioblastoma

Several studies have reported the significant effects of AEBP1-driven tumorigenesis in brain cancer. Glioblastomas (GBM) are one of the most aggressive forms of malignant astrocytoma, where the mean survival rate for GBM patients is 10–12 months [60]. GBM is classified into primary and secondary GBM. While primary GBM develops *de novo* (i.e., without preformation of a less malignant tumor), secondary GBM is preceded by lower-grade astrocytoma [61]. Given that current prognosis of GBM is very poor, a particular study sought to identify novel diagnostic and prognostic biomarkers associated with GBM [42]. A series of transcriptomic analysis studies analyzed genes that are differentially expressed in 16 tumor samples from GBM patients (10 primary and 6 secondary). Genes that were highly expressed in GBM samples, compared to normal brain tissue samples, were identified through real-time quantitative PCR (RT-qPCR). One of the genes that were highly expressed, particularly in primary GBM, was AEBP1. AEBP1 expression was upregulated 4-fold in primary GBM, compared to secondary GBM and other forms of astrocytoma like diffused astrocytoma and anaplastic astrocytoma. These results may render AEBP1 as a potential primary-GBM specific diagnostic marker. Reddy and colleagues [42] suggest that AEBP1 overexpression results in an increased rate of proliferation in primary GBM. AEBP1 was shown to be involved in proliferation due to its high expression in proliferative preadipocytes, compared to terminally differentiated nonproliferative adipocytes [11]. According to Reddy and colleagues [42], these findings collectively underscore the ability of AEBP1 to promote proliferation, which may explain the critical role of AEBP1 in primary GBM.

In line with the study conducted by Reddy and colleagues [42], which examined the effects of AEBP1 overexpression in GBM multiform tumors, Ladha and colleagues [7] sought to analyze the biological significance of AEBP1 overexpression in glioma cells. To study the effect of AEBP1 on tumorigenesis in glioblastoma, endogenous expression of AEBP1 in the astrocyte cell line, U78MG, was silenced using siRNA. Gene expression profiling identified the genes that were affected by AEBP1 silencing, followed by ChIP-chip analysis to confirm which of those genes are targets for AEBP1 binding. Subsequent RT-qPCR data characterized the gene ontology of the targeted genes, revealing a number of cancer-associated genes. Indeed, the analysis revealed that 734 genes were regulated upon AEBP1 silencing. Out of these AEBP1-regulated genes, 27 are related to the cell cycle, 13 are related to differentiation, 27 are related to proliferation, and 21 are related to apoptosis. AEBP1 silencing was accompanied by modulated expression of genes that regulate proliferation of cancer cells (IRS1, EGFR, IL4R, PDGFB, and NRAS) as well as genes that regulate apoptosis (TNFAIP3, TNFAIP8, TNFFRSF10D, TNFSF14, and BIRC5). Among the genes that were downregulated upon AEPB1 silencing is ARNT, a gene involved in protecting cells against hypoxia. Cdc20 and Cdc25C, genes that play a vital role in the cell cycle promoting mitosis, were downregulated whereas CDK6 and MDM2 were upregulated, in cells with silenced AEBP1. ITGB1, FZD8, and NGEF are genes that promote cell differentiation, inducing GBM. While the expression of ITGB1 and FZD8 was upregulated, NGEF expression was downregulated, upon AEBP1 silencing. Furthermore, MTT assay revealed a loss in the proliferative activity in U87MG and U138MG glioma cell lines. The rate of apoptosis was determined in the glioma cell lines by annexin V staining, and TUNEL assay was used to measure DNA fragmentation. Interestingly, apoptosis was upregulated upon AEBP1 silencing in a time-dependent manner in both glioma cell lines [7]. Although AEBP1 silencing influenced the expression of several targets of NF-*κ*B and PI3K signaling in U87MG and U138MG cell lines, there was no apparent effect on NF-*κ*B/p65 expression or localization. Since AEBP1 was not completely depleted, may be low concentrations of AEBP1 were adequate so as not to perturb NF-*κ*B signaling.

Although a direct relationship between AEBP1 and the canonical NF-*κ*B pathway could not be substantiated in the previous study due to only partial depletion of AEBP1, a recent study by Cheng and colleagues [43] shed light on the effects of AEBP1 on NF-*κ*B signaling by treating glioma cell lines with AEBP1-targeted siRNA. Data from the Cancer Genome Atlas (TCGA) revealed high expression of AEBP1 in human gliomas, GBM and low-grade glioma (LGG), compared to noncancerous cells [43]. Kaplan–Meier survival analysis showed that patients with higher expression of AEBP1 have a significantly shorter overall survival (OS) rate. Thus, Cheng and colleagues [43] investigated the effects of AEBP1 silencing in the glioma cell lines, U87MG and U251MG, by transfecting the cells with siAEBP1. CCK-8 assay revealed that AEBP1 silencing caused a significant decrease in the proliferation of the glioma cell lines. Furthermore, ABEP1 silencing decreased the rate of cell invasion and triggered early apoptosis. These results collectively suggest that the targeting of AEBP1 may be effective in attenuating tumor progression in glioma patients. Interestingly, suppression of AEBP1 was associated with a significant decrease in the expression of NF-*κ*B1/p105 (a precursor of NF-*κ*B/p50), along with a decrease in the expression of MMP-2 and Bcl-2, downstream targets of NF-*κ*B. Hence, AEBP1 may promote proliferation and progression of glioma cells via activation of NF-*κ*B pathway and its downstream targets. Moreover, it is suggested that Bax and caspase-3 induce early apoptosis in glioma cells, as AEBP1 silencing resulted in the increased expression of these proteins [43]. This study further affirms the ability of AEBP1 to promote tumor progression via the canonical NF-*κ*B pathway. Notably, the aforementioned studies highlight AEBP1 as an important prognostic biomarker for glioma, and its targeted inhibition may be an effective therapeutic measure for tumor metastasis.

### 2.3. Bladder Cancer

Studies that focused on the tumorigenic effects of AEBP1 in bladder cancer are scarce. Weighted gene coexpression network analysis- (WGCNA-) based studies demonstrated a strong association between AEBP1 expression and tumor progression in bladder cancer. A gene analysis study, using the GSE13507 dataset, highlighted AEBP1 as a potential prognostic biomarker in patients with bladder cancer (BC) [37]. WGCNA analysis of primary BC samples identified AEBP1 as one of the top 50 hub genes associated with tumor node metastasis (TNM) staging in BC patients. A survival analysis of the hub genes, using PROGgenesV2, revealed that low expression of AEBP1 correlated with significantly higher OS rate in BC patients. The effect of AEBP1 on OS was better understood by identifying gene sets enriched in BC samples with high AEBP1 expression. Myogenesis, apical junction, coagulation, and epithelial mesenchymal transition (EMT) were all highly enriched in BC samples with significant AEBP1 expression. High AEBP1 expression was also associated with apoptosis. Importantly, BC samples with high expression of AEBP1 were enriched in gene sets associated with TNF*α* signaling via NF-*κ*B pathway. These results reveal that AEBP1 mediates the progression of BC through NF-*κ*B pathway.

Another study by Di and colleagues [45] screened for genes that are highly expressed and associated with cancer development and proliferation of bladder cancer. By running a WGCNA analysis on the gene chip GSE31685 of bladder cancer, several hub genes were identified as potential biomarkers for bladder cancer, including AEBP1. AEBP1 was correlated with poor prognosis of bladder cancer due to its high expression and association with high degree tumor staging [45]. Furthermore, gene ontology analysis revealed the biological function of AEBP1 in bladder cancer. AEBP1 was associated with several gene ontology items including cell adhesion, organ development, system development, skeletal system development, and multicellular organismal process. NF-*κ*B signaling pathway comprises a rich and complex network, allowing it to regulate a number of biological processes. NF-*κ*B signaling has been shown to regulate a number of the above-mentioned gene ontology items, including cell adhesion, organ development, and skeletal system [59, 62–64]. Hence, a plausible mechanism by which AEBP1 promotes the development of bladder cancer is through the upregulation of tumorigenic processes via NF-*κ*B pathway. Given that the study by Li and colleagues [37] identified the enrichment of NF-*κ*B signaling upon AEBP1 overexpression in their BC dataset, it is plausible to envisage similar results in the BC dataset used in this study. Future studies on the GSE31685 dataset, perhaps using gene enrichment analysis, would yield stronger evidence regarding AEBP1-mediated tumorigenesis via NF-*κ*B pathway, across different BC samples. Nevertheless, as a potential prognostic biomarker, AEBP1 may serve as a therapeutic target in inhibiting the progression of bladder cancer.

Yin and colleagues [46] recently identified a multigene signature associated with the progression of non-muscle invasive bladder cancer (NMIBC) into muscle invasive bladder cancer (MIBC). Two GEO datasets, GSE13507 and GSE120736, were used to analyze mRNA expression of genes associated with disease progression in bladder cancer tissue samples [46]. RT-qPCR analysis presented AEBP1 as one of the six hub genes that were highly expressed in the patient cohort. Although there was no significant difference in AEBP1 gene expression between NMIBC and MIBC samples, the overall upregulation of AEBP1 was correlated with high risk of disease progression and a low OS rate. Furthermore, GSEA identified several canonical pathways enriched in high-risk patients including chemokine signaling pathway, FOXM1 pathway, collagen formation, and extracellular matrix (ECM) remodeling. As we will discuss later, AEBP1 has been repeatedly associated with tumor progression of different types of cancers via stimulation of collagen formation and ECM remodeling. Thus, AEBP1 may promote the progression of NMIBC to MIBC through activation of these pathways. Another possible mechanism by which AEBP1 promotes disease progression is EMT. AEBP1 was also shown to promote EMT in gastric cancer and colorectal cancer via activation of NF-*κ*B pathway [47, 49]. These results suggest that the augmented expression of AEBP1 may accelerate the progression of disease to MIBC by triggering collagen formation, ECM remodeling, and EMT, with a possible mediation via NF-*κ*B pathway. Importantly, AEBP1 may be used as a diagnostic biomarker for tumor progression in bladder cancer and may be a potential target for individualized therapies for patients with high risk of MIBC.

### 2.4. Gastric Cancer

Given that the function of AEBP1 in gastric cancer is rendered largely unknown, a particular study focused on the potential mechanisms by which AEBP1 promotes progression of gastric cancer (GC) cells [47]. In an immunohistochemistry study, AEBP1 was shown to be highly expressed in human GC tissue and cell lines, compared to adjacent normal tissue. Similar results were found upon detection of mRNA expression of AEBP1. Consequently, upregulation of AEBP1 expression in GC cells was accompanied by poor prognosis and low OS rate in patients with GC. AEBP1 silencing caused a significant suppression of proliferation and colony formation in the GC cell lines, MGC803 and XN0422. Furthermore, AEBP1 silencing caused a significant inhibition of GC cell migration, invasion, and metastasis compared to control cells. Interestingly, an *in vivo* experiment, using subcutaneous xenograft model in nude mice, demonstrated a decrease in weight and size of xenograft tumors upon AEBP1 silencing. These results collectively indicate that AEBP1 might possess oncogenic activities in GC. Importantly, it was revealed that AEBP1 enhances GC cell progression due to its ability to promote EMT of GC cells via activation of NF-*κ*B pathway. AEBP1 silencing resulted in the reduction of NF-*κ*B/p65 and phosphorylated NF-*κ*B/p65 (p-NF-*κ*B/p65) levels in GC cells. These findings were also shown *in vivo* using xenograft tumors. Moreover, AEBP1 silencing was accompanied by impaired nuclear translocation of NF-*κ*B/p65 and inhibited expression of CXCR4 and ICAM-1, downstream targets of NF-*κ*B signaling that are involved in tumor growth, invasion, and metastasis. Furthermore, AEBP1 silencing caused an upregulation of I*κ*B*α*, resulting in the attenuation of NF-*κ*B signaling and the subsequent suppression of EMT. TNF*α* treatment, a means of inducing NF-*κ*B signaling, in GC cells with silenced AEBP1 reestablished EMT. This further underscores the ability of AEBP1 to positively regulate the canonical NF-*κ*B pathway. Therefore, targeting AEBP1 could prove to be an effective therapeutic approach to treat GC patients.

### 2.5. Colorectal Cancer

Despite the discovery of effective treatment strategies for colorectal cancer (CRC), the typical survival rate of patients is a mere five years. CRC is the third most common type of malignant tumors in the world; thus, identifying prognostic biomarkers that could improve the diagnosis of the disease and lead to targeted therapy is of great importance. Li and colleagues [48] investigated the potential role of AEBP1 in tumor progression of CRC by analyzing CRC tissues from randomly selected patients. Results revealed high expression of AEBP1 in the nucleus and cytoplasm, compared to normal colonic mucosal tissue. High levels of AEBP1 were associated with the presence of lymph node metastasis and TNM staging. It was further observed that high expression of AEBP1 correlated with poor OS rate and disease-free survival (DFR) in CRC patients. The association of AEBP1 with lymph node metastasis may explain the poor OS rate and the manifestation of high TNM stages III and IV. Interestingly, the effect of AEBP1 on apoptosis and cell proliferation was studied using human CRC HT-29 cell line transfected with miR-214, an AEBP1 depletion siRNA. Upon depletion of AEBP1, cells were treated with the chemotherapeutic drug oxaliplatin. Suppression of AEBP1, coupled with oxaliplatin treatment, caused an increase in apoptosis and a significant decrease in the rate of cell proliferation. Importantly, cotransfection of a miR-214 mimic and a luciferase reporter gene resulted in the decrease of the reporter gene activity by 30%, indicating that miR-214 is an upstream target gene for AEBP1. Identifying miR-214 as a negative regulator of AEBP1 may lead to the therapeutic targeting of AEBP1 and suppression of tumor progression in CRC patients [48].

The aforementioned study was the first to investigate the role of AEBP1 in the development and progression of CRC. Subsequently, whether NF-*κ*B pathway plays a role in AEBP1-mediated tumorigenesis of CRC was investigated. Xing and colleagues [49] attempted to elucidate the signaling pathways by which AEBP1 enhances the metastatic features in colorectal adenocarcinoma (COAD). To this end, tissue samples from 12 COAD patients (6 females and 6 males) were analyzed for AEBP1 expression levels. Compared to adjacent normal colon tissue, mRNA and protein levels of AEBP1 were significantly higher in COAD tissue samples. Furthermore, COAD cell lines (DLD-1, LoVo, HCT116, and SW1116) exhibited significantly higher levels of mRNA and protein levels, compared to normal colon epithelial cells (NCM460). These results demonstrated that AEBP1 is differentially upregulated in COAD patient samples, compared to normal tissue and cells. A subsequent immunohistochemical experiment, using 101 COAD patient tissue samples, highlighted the fact that AEBP1 overexpression in COAD is associated with advanced clinicopathological features and poor OS. Upregulation of AEBP1 was correlated with tumor size, histologic differentiation level, lymph node metastasis, and tumor staging. Kaplan–Meier analysis revealed that patients with higher levels of AEBP1 had a poorer OS rate. Importantly, multivariate COX expression analysis identified AEBP1 as one of the independent prognostic factors for OS in COAD patients [49]. To evaluate if AEBP1 overexpression is indeed directly correlated with tumorigenesis in COAD, the effects of downregulating AEBP1 *in vitro* and *in vivo* were assessed. Depleting AEBP1 by transfecting LoVo and DLD-1 cells with AEBP1-specific siRNA oligos resulted in decreased cell proliferation and colony formation *in vitro*. Interestingly, a wound-healing assay and a transwell invasion assay showed that AEBP1 silencing inhibited the migratory and invasion abilities of both LoVo and DLD-1 cells. On the contrary, inducing AEBP1 overexpression in SW1116 cells via a pcDNA3.1(+)-AEBP1 vector augmented cell proliferation, migration, and invasion. Taken together, these observations indicate that AEBP1 promotes the progression of COAD, and its inhibition may prove affective in attenuating such metastatic features. Intriguingly, a xenograft mouse model was used to study the effect of AEBP1 on COAD tumorigenesis *in vivo*. Athymic BALB/c nude mice were subcutaneously injected with DLD-1 cells transfected with AEBP1 short hairpin RNA (Lv-sh-AEBP1 #2) or scramble (control). Tumor weight and volume in the DLD-1-Lv-sh-AEBP1 #2 mouse group were significantly lower than those in the control mouse group. Analysis of Ki67 protein expression for cell proliferation reported a significantly decreased rate of proliferation in the AEBP1-silenced mouse group, compared to the control group. Remarkably, the number of metastatic lung nodules in the DLD-1-Lv-sh-AEBP1 #2 group was reduced compared to the control group. These results demonstrate that AEBP1 promotes the proliferative and metastatic abilities of COAD *in vivo* [49]. Hence, targeting AEBP1 in this mouse model may be an effective initiative in the future development of a therapeutic agent for patients with COAD.

To understand how AEBP1 promotes the progression of COAD cells, the relationship between AEBP1 and EMT-related genes (E-cadherin, MMP-2, vimentin, and TWIST) was investigated. EMT is the process by which epithelial cells lose cell-cell adhesion and acquire migratory and invasive abilities [65]. Studies suggest that overactivation of EMT leads to tumor metastasis and progression [66, 67]. RT-qPCR analysis revealed that mRNA levels of MMP-2, vimentin, and TWIST were reduced in AEBP1-silenced DLD-1 and LoVo cells. In contrast, AEBP1 overexpression in SW1116 cells enhanced MMP-2, vimentin, and TWIST expression. Similar results were observed at the protein level in DLD-1, LoVo, and SW1116 cells using Western blot analysis. Consistently, a coexpression analysis of 331 COAD samples using the ChIPBase v2.0 demonstrated a positive relationship between AEBP1 level and the expression of MMP-2, vimentin, TWIST, ZEB1, ZEB2, and SNAIL1, inducers of tumor growth, invasion, and metastasis. These results strongly indicate that AEBP1 enhances the metastatic and pathological features of COAD cells by augmenting the expression of genes involved in the EMT process [49]. There was a weak correlation between AEBP1 and E-cadherin. Neither AEBP1 silencing nor its overexpression in SW1116 cells had any significant effect on mRNA expression of E-cadherin. However, E-cadherin expression was upregulated in AEBP1-silenced DLD-1 and LoVo cells, while it was downregulated in AEBP1-overexpressing SW1116 cells, suggesting that AEBP1 influences E-cadherin at the protein level. E-cadherin is a cell-adhesion molecule that plays a role in cell polarity and adhesion [68]. The loss of E-cadherin triggers the EMT process and consequently promotes tumorigenesis [69, 70]. Collectively, these results indicate that AEBP1 overexpression induces the EMT process by suppressing E-cadherin expression, thereby enhancing tumor progression in COAD. Interestingly, AEBP1 was shown to regulate NF-*κ*B signaling pathway in COAD cells. Western blot analysis revealed that NF-*κ*B/p65 and p-NF-*κ*B/p65 levels significantly decreased upon AEBP1 silencing and increased upon AEBP1 overexpression. Furthermore, an important study using BAY 11-7082, an IKK/NF-*κ*B inhibitor, indicated that AEBP1 influences the expression of MMP-2, vimentin, TWIST, and E-cadherin via NF-*κ*B pathway. SW1116 cells overexpressing AEBP1, treated with BAY 11-7082, resulted in decreased levels of MMP-2, vimentin, and TWIST. However, E-cadherin levels were augmented. Notably, BAY 11-7082 significantly decreased colony formation and invasion abilities of SW1116 cells with AEBP1-overexpression. Thus, AEBP1 has been shown not only to mediate that expression of EMT-related components through NF-*κ*B signaling, but also to promote the progression and metastatic features of COAD, *in vitro* and *in vivo*, via the canonical NF-*κ*B pathway.

In a very recent study, Yorozu and colleagues [71] demonstrated that AEBP1 upregulation in endothelial cells stimulates tumor angiogenesis in CRC [71]. Interestingly, AEBP1 expression in human umbilical vein endothelial cells (HUVECs) was shown to be increased by tumor-conditioned medium derived from CRC cells as well as by direct coculture with CRC cells. Furthermore, AEBP1 silencing caused significant suppression in the proliferation, migration, and *in vitro* tube formation by HUVECs. Using a xenograft model, AEBP1 silencing was accompanied by inhibited tumorigenesis and microvessel formation. Moreover, AEBP1 silencing in HUVECs led to a significant decrease in the expression of genes that are critically implicated in angiogenesis including aquaporin 1 (AQP1) and periostin (POSTN). The findings of this study clearly indicate that AEBP1 is a crucial positive regulator of angiogenesis in CRC through its ability to modulate the expression of angiogenesis-inducing genes.

### 2.6. Ovarian Cancer

Intrinsic and acquired chemoresistance pose a great challenge for patients with ovarian carcinoma who do not respond to standard chemotherapy. Therefore, identifying biomarkers that can predict negative patient outcomes may assist in the development of more personalized treatment strategies.

In line with this challenge, Cheon and colleagues [50] sought to identify gene signatures in serous ovarian cancer (SOC) that are associated with metastasis and poor survival outcome. Microarray analyses of high-grade (TCGA dataset), advanced-stage (GSE26712), and primary (GSE51088) ovarian carcinoma samples revealed a 10-gene signature correlated with poor OS rate [50]. Interestingly, AEBP1 was reported to be highly expressed in the three datasets, suggesting that AEBP1 is highly correlated with poor OS in patients with SOC. A Pearson correlation test exhibited a high correlation in the expression of the ten genes, suggesting that the genes play a similar biological role. Given that the genes are known to be localized in the extracellular matrix and play a role in cell adhesion and collagen remodeling, the collagen-remodeling abilities of AEBP1 may be associated with poor OS and metastasis of SOC. Furthermore, the expression of collagen-remodeling genes was highly evident in metastatic serous ovarian tumors. Indeed, mRNA expression of such genes was reported in primary and metastatic ovarian cancer from three ONCOMINE datasets (Anglesio, Bittner, and Tothill). ONCOMINE is a cancer microarray database and integrated data-mining platform aimed at facilitating discovery from genome-wide expression analyses [72]. It was demonstrated that the expression of collagen-remodeling genes was significantly higher in metastatic samples, compared to primary samples, across the three datasets. These findings further support the involvement of the collagen-remodeling genes in the progression of SOC.

Additionally, ingenuity pathway analysis (IPA) revealed that the signature genes were regulated by TGF*β* signaling pathway, including several other transcription factors implicated in that pathway (TGF-*β*2, TGF*β*3, SMAD3, and SMAD7). The reported results showed that TGF*β* signaling pathway did not directly regulate AEBP1 expression. However, a follow-up experiment using the ovarian cancer cell line, OVCAR3, demonstrated that treating cells with TGF*β*1 increased mRNA level of AEBP1. Furthermore, AEBP1 expression was abrogated when cells were treated with the TGF*β*1 receptor inhibitor, A83-01, followed by TGF*β*1 treatment. These results collectively suggest that TGF*β* signaling pathway may be indirectly involved in regulating AEBP1 expression in SOC. Interestingly, IPA identified NF-*κ*B complex as part of the molecular network in TGF*β* signaling. Since we have previously highlighted the positive role that AEBP1 plays in regulating NF-*κ*B pathway, it is conceivable to speculate that TGF*β* may indirectly regulate AEBP1 in SOC through crosstalk with NF-*κ*B pathway. Importantly, this study sheds light on AEBP1 as a potential therapeutic target to attenuate poor survival outcome in women with SOC [50].

Ovarian cancer is the most lethal form of gynecological cancer, where most of the cases are diagnosed at an advanced stage due to inaccurate early screening methods. Thus, a great importance is placed on the identification of biomarkers to help predict prognosis and development of ovarian cancer. A WGCNA-based study revealed several hub genes that are highly associated with grade and tumor stage in SOC [21]. Analysis of three ovarian datasets from SOC patients (GSE26193, GSE9891, and TCGA) identified AEBP1 as one of the top 25 genes correlated with tumor staging of SOC. In fact, AEBP1 was shown to be the 4^th^ top gene in the stage-related module. To validate the correlation between the hub genes network and tumor staging in SOC, Sun and colleagues [21] determined the statistical significance using Kruskal–Wallis tests and further used five independent validation SOC datasets (GSE49997, GSE17260, TCGA.RNASeqV2, GSE20565, and PMID15897565) to determine if the module eigengene expression, a statistical analysis approach to study relationships among coexpression modules, was universal across SOCs. The analysis revealed a strong positive correlation between the expression of the hub genes and different tumor stages (stages I–IV). In other words, AEBP1 expression positively correlated with the progression of tumor stage. The results obtained across several SOC datasets emphasize the role of AEBP1 as a metastasis-promoting biomarker. Furthermore, there was a significant positive correlation between AEBP1 expression and four validation datasets (GSE49997, GSE17260, TCGA.RNASeqV2, and PMID15897565), demonstrating that the involvement of AEBP1 in the development of SOC is universal. The interactions among coexpressed hub genes within the datasets were examined using Search Tool for the Retrieval of Interacting Genes (STRING). Intriguingly, AEBP1 demonstrated a strong interaction with MMP-2, with a combined score of above 600 in the high-quality STRING subnetwork. In line with these findings, gene ontology and pathway enrichment analysis identified ECM organization as the top enriched term in the stage-related module, including AEBP1. Furthermore, the top enriched terms on Kyoto Encyclopedia of Genes and Genomes (KEGG) pathway analysis were “focal adhesion” and “ECM-receptor interaction.” Consequently, AEBP1 may contribute to tumor staging and development of SOC due to its strong interaction with MMP-2 and ECM components. Crosstalk between ECM and NF-*κ*B pathway was implicated in ovarian cancer, thereby inducing a sequence of inflammatory responses and upregulation of biological processes that promote metastasis [73, 74]. Taken together with the study conducted by Cheon and colleagues [50], upregulation of ECM components and NF-*κ*B pathway due to AEBP1 overexpression could serve as a plausible module for AEBP1-mediated tumorigenesis in ovarian cancer. In conclusion, AEBP1 could prove to be a reliable early diagnostic marker and its targeting could prove to be an effective therapeutic intervention in patients with SOC.

### 2.7. Skin Cancer

We have repeatedly underscored the role of AEBP1 in augmenting the metastatic abilities of cancer cells through the upregulation of EMT. In melanoma patients, AEBP1 expression was reported in cancer-associated fibroblasts (CAFs). CAFs are stromal cells that surround the tumor microenvironment and undergo EMT, thereby promoting tumor progression and metastasis [51]. Sasaki and colleagues [51] demonstrated that specific CAF phenotypes, present in cutaneous malignant tumors, highly influence the migratory ability of cancer cells by promoting EMT. To characterize the profile of CAFs, stromal fibroblasts from tumors of patients with basal cell carcinoma (BCC), squamous cell carcinoma (SCC), and malignant melanoma (MM) were tested for CAF- and EMT-related proteins. Immunohistochemical staining of tumors revealed the expression of several CAF- and EMT-related proteins in the tumor microenvironment, including AEBP1. It is worthy of note that AEBP1 was not expressed in BCC and SCC. However, immunostaining intensity revealed a low expression of AEBP1 in CAFs associated with MM tumors, presenting AEBP1 as a possible CAF marker. Previous studies have established that MM is associated with high frequency of lymph node metastasis [75], suggesting that CAF-related markers may serve as predictors of tumor progression and metastasis. Although Sasaki and colleagues [51] have identified AEBP1 as a possible CAF biomarker, the specific role of AEBP1 in this tumor microenvironment was not characterized. Given that CAFs promote tumorigenesis through EMT, AEBP1 may contribute to the CAF phenotype by the induction or upregulation of EMT-related genes. The role that AEBP1 plays in augmenting the expression of EMT-related proteins via NF-*κ*B pathway in bladder, gastric, and colorectal cancers was previously underscored [37, 47, 49]. AEBP1 may display similar effects in CAFs, enhancing the metastatic features and proliferative abilities of cancer cells within the tumor microenvironment. Nevertheless, the presence of AEBP1 in CAFs was reported in a limited patient sample. Thus, a larger study may further substantiate AEBP1 as a critical CAF-related biomarker that promotes tumor progression and invasiveness in cutaneous malignant tumors.

Although the exact role of AEBP1 in CAFs in melanoma patients requires further analysis, Hu and colleagues [52] conducted a detailed experiment to examine the potential role of AEBP1 in acquired drug resistance to BRAF inhibition of melanoma via NF-*κ*B pathway. Mutations in the protooncogene BRAF constitute about half of melanoma cases worldwide [76]. Although there are a number of effective BRAF inhibitors, including vemurafenib (PLX4032), acquired drug resistance has been significantly reported in patients during initial treatment. To characterize the underlying mechanisms behind drug resistance in metastatic melanoma, Hu and colleagues [52] used PLX4032-resistant cells lines, produced by chronically exposing Mel-CV melanoma cells to PLX4032. Upon generation of PLX4032-resistant melanoma cells (Mel-CVR18 and Mel-CVR21), cDNA microarray and RT-qPCR analysis of these cells revealed a significant upregulation of AEBP1, compared to Mel-CV cells [52]. Interestingly, AEBP1 knockdown in Mel-CVR cells, followed by treatment with PLX4032, resulted in a significant decrease in cell viability, compared to control cells. An *in vivo* experiment revealed the importance of AEBP1 by using nude mice injected with Mel-CVR cells with AEBP1 knockdown, followed by treatment with PLX4032. Mice injected with Mel-CVR cells with AEBP1 knockdown had significantly smaller tumors than mice injected with unmanipulated Mel-CVR cells. These results reveal that the effects of AEBP1 extend to *in vivo* settings, which suggests that targeting AEBP1 in melanoma patients subjected to BRAF inhibitors may be a promising therapeutic step.

Previous studies have demonstrated that activation of NF-*κ*B pathway confers a protective activity on melanoma cells subjected to BRAF inhibition [77]. Similarly, Hu and colleagues [52] reported that AEBP1 mediated the activation of NF-*κ*B pathway in Mel-CVR cells. Mel-CVR cells were reported to have lower I*κ*B*α* expression and higher NF-*κ*B/p65 activity, compared to Mel-CV cells [52]. AEBP1 knockdown downregulated the transcriptional activity of NF-*κ*B/p65. To determine if AEBP1 influences NF-*κ*B signaling directly or through a mediator, the effects of CREB on NF-*κ*B activation were investigated upon AEBP1 knockdown. CREB was chosen as a potential mediator due to its reported relationship with AEBP1 in Mel-CVR cells, which will be discussed in a subsequent section. Interestingly, Mel-CVR cells transfected with a mutant CREB revealed no significant upregulation of NF-*κ*B/p65 transcriptional activity, compared to Mel-CVR cells with wild-type CREB. These results indicate that AEBP1 is a vital downstream mediator in the activation of the canonical NF-*κ*B pathway. It was shown that the knockdown of AEBP1 resulted in a marked decrease in cell viability. Further inhibition of NF-*κ*B/p65 activity, followed by treatment with PLX4032, resulted in decreased cell viability of Mel-CVR cells. These results strongly suggest that the canonical NF-*κ*B pathway is a critical mediator of acquired drug resistance to BRAF inhibition in melanoma. Consequently, targeting AEBP1 may prove to be an effective therapy for drug resistance in melanoma patients subjected to BRAF inhibitors.

Another form of skin cancer where AEBP1 was demonstrated to be an effective therapeutic target is BCC. One clinical variant of human BCC is characterized by the differentiation along the hair follicle. Morgan and colleagues [53] sought to identify genes that are differentially expressed during the hair growth cycle in BCC. The hair cycle is defined by three distinct phases that the hair follicle is subjected to, including cyclical growth (anagen), apoptosis (catagen), and rest (telogen). They theorized that targeting exogenous modulators that promote anagen differentiation in BCC could lead to the exhaustion of BCC cancer stem cells [53]. RT-qPCR analysis of human BCC tissue samples demonstrated a significant upregulation of AEBP1 in the telogen phase. Immunolabeling of BCC tissue further illustrated AEBP1 overexpression in telogen. Morgan and colleagues [53] stated that one of the factors that hinder anagen BCC differentiation is the overexpression of telogen modulators, including AEBP1. A decrease in BCC differentiation would enable the progression of BCC cancer stem cells. It was shown that the administration of TGF-*β*2 and Noggin to BCC cell cultures enhanced the expression of DLX3, an anagen master regulator, while the expression of telogen-associated genes was suppressed. Therefore, this study proposes that promoting anagen differentiation of the hair follicle in BCC could prove to be an effective BCC therapeutic method. In theory, inhibiting the telogen phase of the hair cycle would also stimulate the differentiation of BCC and thereby the exhaustion of BCC cancer stem cells. Therefore, a probable therapeutic target for patients with BCC is AEBP1. Inhibitors of AEBP1 may prove to be effective therapeutic agents for blocking the progression of BCC in patients.

## 3. AEBP1 Promotes Tumorigenesis via PI3K-Akt Signaling Pathway

A large number of studies investigated the relationship between PI3K-Akt pathway and cancer. Phosphatidylinositol 3 kinases (PI3Ks) and protein kinase B (Akt) are a group of enzymes that are known to regulate several cellular processes including cell growth, proliferation, survival, migration, apoptosis, and differentiation [78, 79]. Hyperactivation of PI3K-Akt pathway has been implicated in many types of human cancers, consequently leading to the uncontrollable activation of the aforementioned cellular processes [80, 81]. The abnormal and sustained activation of cell growth, cell proliferation, and migration is a well-known characteristic that prompts carcinogenesis and tumor progression. A few studies have reported a positive correlation between AEBP1 expression and activation of PI3K-Akt pathway in different types of cancers. In this section, we will discuss the relationship of AEBP1 and PI3K-Akt pathway in melanoma, breast cancer, glioblastoma, and leukemia (Table 1).

As discussed earlier, AEBP1 possesses the ability to promote tumor progression in PLX-4032-resistant melanoma via NF-*κ*B pathway [52]. However, NF-*κ*B pathway is not the only pathway that is influenced by AEBP1 overexpression in melanoma patients with resistance to BRAF inhibition. A series of genomic analyses demonstrated that AEBP1 expression in Mel-CVR cells is regulated by PI3K-Akt-CREB-NF-*κ*B pathway [52]. CREB is a downstream regulator in PI3K-Akt signaling pathway [82, 83]. Luciferase assay revealed positive transcriptional activity of wild-type CREB binding region (CREB-BR) in Mel-CVR and Mel-CV cells. However, mutant CREB-BR and deletion of CREB-BR demonstrated no transcriptional activity in the cell lines. Interestingly, transcriptional activity of CREB-BR was higher in Mel-CVR cells compared to the control cell line. While CREB-BR augmented AEBP1 transcriptional activity in Mel-CVR cells, CREB knockdown led to a decrease in AEBP1 mRNA level and a decrease in cell viability. Similar experiments suggested that PI3K-Akt pathway further influences AEBP1 expression in PLX4032-resistant melanoma cells. Phosphorylated Akt (p-Akt) was higher in Mel-CVR cells, compared to Mel-CV control cells. Moreover, treating Mel-CVR cells with LY294002, a PI3K-Akt inhibitor, caused a significant decrease in the protein levels of p-CREB and AEBP1 [52]. These results collectively indicate that PI3K-Akt-CREB pathway plays a critical role in AEBP1 overexpression, which in turn stimulates acquired drug resistance to BRAF inhibition in melanoma patients. Further experimental analysis suggested that PI3K-Akt-CREB pathway interacts with NF-*κ*B pathway in Mel-CVR cells [52]. Mel-CVR cells treated with Myr-Akt, a constitutively active form of Akt, caused a marked increase in NF-*κ*B/p65 activity. These results indicate that a crosstalk may exist between PI3K-Akt-CREB pathway and the canonical NF-*κ*B pathway. We speculate that this crosstalk may be mediated by AEBP1. Since PI3K-Akt was shown to augment AEBP1 expression, and since AEBP1 mediates the activation of the canonical NF-*κ*B pathway [52], AEBP1 might act as the mediator between the two signaling pathways. This proposed module could lead to the enhanced BRAF inhibitor resistance in melanoma patients. Thus, inhibiting the mediator, AEBP1, may prove to be an effective and practical therapeutic method in combating PLX4032-resistant melanoma. Based on these findings, it was concluded that PI3K-Akt-CREB-AEBP1-NF-*κ*B pathway may be presented as a novel pathway whose activation promotes acquired resistance to BRAF inhibitors in melanoma. The molecular network of the proposed PI3K-Akt-CREB-AEBP1-NF-*κ*B pathway is illustrated in Figure 2.

AEBP1 was further shown to influence Akt expression and activity in mammary gland tumorigenesis [28]. Treating HC11 mammary epithelial cells with AEBP1^TG^ macrophage-supernatant caused pAkt upregulation. On the contrary, treating HC11 cells with an AEBP1^−/−^ macrophage-supernatant resulted in a dramatic downregulation of pAkt. Hence, macrophage AEBP1 expression could augment the activity of PI3K-Akt. Interestingly, AEBP1 was shown to upregulate the activity of pAkt through TNF*α*. The addition of anti-TNF*α* antibody dramatically decreased pAkt activity, indicating that macrophage AEBP1 may enhance Akt activation via TNF*α* signaling. AEBP1 is therefore reported to promote tumor progression in mammary epithelial cells through upregulation of TNF*α* secretion, thereby augmenting the Akt-mediated survival signal [28]. Given that AEBP1 is observed to promote the activity of both NF-*κ*B/p65 and Akt via TNF*α* signaling [28], this may indicate that AEBP1 mediates the crosstalk between PI3K-Akt pathway and the canonical NF-*κ*B pathway in mammary gland hyperplasia.

As mentioned above, AEBP1 was shown to maintain the survival and proliferation of U138MG glioma cells [42]. In line with these findings, Sinha and colleagues [44] revealed the possible mechanisms by which AEBP1 may promote tumorigenesis in glioma cells. The effect of depleting AEBP1 in U138MG cells revealed a significant effect on PI3KCB, a specific PI3K enzyme. AEBP1 silencing was shown to lead to the downregulation of PI3KCB activity at the mRNA and protein levels. The PI3KCB promoter was demonstrated to possess an AEBP1 binding motif [44], which was also demonstrated by Ladha and colleagues [7] using ChIP-chip analysis. Consequently, this could explain the stimulatory effect of AEBP1 on PI3KCB. To further confirm the observed positive relationship between AEBP1 and PI3KCB, the Cancer Genome Atlas Glioblastoma Multiform (TCGA-GBM) dataset was used to measure the median expression of AEBP1 and PI3KCB. The TCGA-GBM dataset consists of the genomic and transcriptomic analysis of 206 glioblastoma samples, which seek to define genetic mutations and alterations that trigger carcinogenesis [84]. The results reported a strong positive correlation between AEBP1 and PI3KCB. Since PI3Ks are a family of enzymes involved in the activation of signaling pathways responsible for cell proliferation, growth, and survival [85], AEBP1-mediated positive regulation of PI3KCB may be one factor that leads to the progression of glioma.

In addition, an immunofluorescence analysis revealed an inverse relationship between PI3KCB and *γ*H2AX foci [44]. *γ*H2AX foci assay is an efficient and sensitive experimental approach that is employed for the detection of double-strand breaks, a form of DNA damage. AEBP1 silencing in U138MG cells led to a temporal decrease of PI3KCB and an increase of *γ*H2AX foci. Additionally, PI3KCB overexpression in AEBP1-depleted glioma cells inhibited the accumulation of *γ*H2AX foci. Since PI3KCB plays an important role in maintaining genomic integrity [86], Sinha and colleagues [44] suggest that the decrease in PI3KCB leads to an increase in double-strand breaks in the nuclei of AEBP1-depleted glioma cells. These results collectively indicate that AEBP1 plays a significant role in gliomagenesis, possibly through the PI3K-Akt pathway. Hence, AEBP1 downregulation may be an effective therapeutic target for the treatment of GBM.

Li and colleagues [54] identified AEBP1 as one of the abnormally overexpressed genes that may play a significant role in the pathogenesis of childhood acute lymphoblastic leukemia (cALL). Series Test of Cluster (STC) analysis revealed a high expression of AEBP1 in two cALL datasets at the relapse stage (GSE28460 and GSE60926), while AEBP1 expression was significantly decreased in the posttreatment cALL dataset (GSE67684). These results suggest that AEBP1 plays a critical role in the relapse of cALL and may serve as an important diagnostic biomarker for cALL. To understand the potential pathogenic effects of AEBP1, a gene ontology functional enrichment analysis was used in the relapse-stage cALL datasets, where AEBP1 was observed to be overexpressed [54]. The gene ontology functional enrichment analysis identified several genes that were coexpressed due to AEBP1 overexpression in the cALL datasets. Of the 200 identified AEBP1 coexpressed genes, nine genes (TCL1A, THBS1, TLR4, PDGFC, CSF3R, ITGB3, IL6R, ITGA2B, and VEGFA) were reported to enrich the PI3K-Akt pathway. This suggests that AEBP1 may promote the relapse of cALL by augmenting the activity of PI3K-Akt signaling pathway, via upregulation of the aforementioned AEBP1 coexpressed genes. This study sheds light on the potential pathogenic role of AEBP1 in cALL via stimulation of PI3K-Akt pathway. However, future *in vitro* analyses using cALL cell lines and PI3K inhibitors could provide more substantial evidence on the relationship between AEBP1 and PI3K-Akt in cALL relapse.

## 4. AEBP1 Promotes Tumorigenesis via Sonic Hedgehog (Shh) Signaling Pathway

Sonic hedgehog (Shh) signaling is one of the key pathways involved in embryonic development, organogenesis, and morphogenesis [87, 88]. Furthermore, sonic hedgehog (Shh) signaling has been implicated in the development and metastasis of different types of cancers, including breast cancer [89–91].

Experimental findings from our laboratory illustrated the role of AEBP1 in promoting mammary cell hyperplasia via sonic hedgehog (Shh) signaling [28]. Overexpression of stromal macrophage AEBP1 was reported to upregulate the activation of NF-*κ*B signaling in HC11 mammary epithelial cells [28]. Given that NF-*κ*B signaling was reported to regulate sonic hedgehog (Shh) signaling in macrophages [92–94], we previously assessed the ability of AEBP1 to induce sonic hedgehog (Shh) signaling [28]. Treating HC11 cells with AEBP1^TG^ macrophages resulted in the upregulation of sonic hedgehog (Shh) expression, along with Gli1 and Bmi1 mRNA levels 4- and 2-fold, respectively. *Gli1* and *Bmi1* are sonic hedgehog (Shh) target genes and their aberrant expression is implicated in tumorigenesis. *Gli1* and *Bmi1* are protooncogenes whose products were shown to promote stem cell proliferation, differentiation, and development [46, 95]. These results suggest that AEBP1-mediated upregulation of sonic hedgehog (Shh) signaling, Gli1, and Bmi1 promotes mammary gland development and tumorigenesis. Furthermore, the dual effect of AEBP1 on the canonical NF-*κ*B and sonic hedgehog (Shh) signaling pathways may indicate that AEBP1 induces a synergistic effect that consequently leads to inflammation and metastasis (Figure 2). Importantly, this study paves the way for future studies to investigate the effect of AEBP1 on sonic hedgehog (Shh) signaling in various types of cancers (Table 1). TSHZ2 (zinc-finger homeobox protein) was shown to be a transcriptional repressor that is downregulated in breast cancer cells and to exert negative regulation on Gli1 activity in normal and immortalized mammary gland duct epithelium [41]. Riku and colleagues [41] demonstrated that TSHZ2, along with CtBP as a corepressor complex, represses the expression of Gli1 and AEBP1 in MCF-7 breast cancer cells, and TSHZ2 knockdown leads to enhanced expression of Gli1 and AEBP1 in primary human mammary epithelial cells (HMECs) and TSHZ2-expressing invasive mammary ductal carcinoma. Intriguingly, Gli1 was shown to impose positive regulation on AEBP1 expression in HMECs and MCF-7 cells [40, 41]. Indeed, the expression of AEBP1, a Gli1-regulated factor, was significantly increased in invasive mammary ductal carcinoma. It was concluded that the suppressed expression of TSHZ2 in invasive mammary ductal carcinoma is accompanied by enhanced Gli1 transcriptional activity, leading to increased AEBP1 expression. Based on these findings, we postulate a positive feedback loop between AEBP1 and Gli1, further confirming a positive interplay between AEBP1 activity and sonic hedgehog (Shh) signaling. The main regulatory effects of AEBP1 on cancer-related proteins involved in hedgehog (Shh) pathway are illustrated in Figure 1.  Figure 2 depicts the positive interplay between AEBP1 and hedgehog (Shh) signaling.

## 5. AEBP1 Promotes Tumorigenesis via p53 Signaling Pathway

Multiple cALL datasets revealed the ability of AEBP1 to promote the relapse of cALL via PI3K-Akt pathway [54]. Li and colleagues [54] sought to analyze AEBP1 expression in cALL *in vitro* and further investigate the effects of silencing AEBP1 in cALL. AEBP1 expression was upregulated in multiple leukemia cell lines including Jurkat, Nalm-6, and Raji cells, compared to normal cell line PBMCs. CCK-8 assay revealed that AEBP1 silencing markedly decreased the proliferative rate of Jurkat cells. AEBP1 silencing further caused a dramatic decrease in the percentage of Jurkat cells at the S phase and an increase in the percentage of Jurkat cells in the G1 phase, demonstrating that AEBP1 silencing causes cell cycle arrest in Jurkat cells. Moreover, AEBP1 silencing caused a decrease in Bcl-2 expression in Jurkat cells. Intriguingly, AEBP1 silencing resulted in an upregulation of caspase-3, caspase-7, and Bax, leading to an accelerated rate of apoptosis in Jurkat cells. These effects, caused by AEBP1 silencing, were accompanied by modulation of p53 and PI3K-Akt signaling in Jurkat cells, suggesting that AEBP1 silencing promotes apoptosis via a p53-and PI3K-Akt-dependent mechanism. Collectively, these findings suggest that AEBP1 promotes cell survival and blocks apoptosis by targeting p53 and PI3K-Akt signaling pathways (Table 1).

The inhibitory effect of AEBP1 on p53 was further demonstrated in GBM [7]. AEBP1 was reported to be overexpressed in the astrocyte cell line, U78MG, and its overexpression was believed to be indicative of tumor progression [7]. AEBP1 silencing, using an AEBP1-targeted siRNA, influenced the expression of multiple genes involved in regulating cell growth, proliferation, differentiation, and apoptosis. Interestingly, *TP53*, the gene that encodes p53 protein, was one of the genes that were upregulated due to AEBP1 silencing. Since p53 is a potent tumor suppressor that regulates numerous processes including apoptosis, cell cycle arrest, and DNA repair [96, 97], its upregulation in AEBP1-silenced U78MG cells is considered a therapeutic advantage. Thus, targeting AEBP1 may attenuate the pathogenic and metastatic activities by upregulation of p53, thereby leading to a novel approach in treating patients with GBM (Table 1). The main regulatory effects of AEBP1 on cancer-related proteins involved in p53 pathway are illustrated in Figure 1.  Figure 2 displays the negative regulatory role of AEBP1 on p53 signaling.

## 6. AEBP1 Regulates Cell Growth and Proliferation via Parthanatos (PARP-1) Signaling Pathway

Since AEBP1 silencing in U138MG glioma cells was shown to lead to cell death [42], a recent study sought to explore the mechanism by which cell death is induced upon depletion of AEBP1 [44]. Interestingly, this study demonstrated that cell death in AEBP1-depleted U138MG cells occurs in a caspase-independent manner. Upon AEBP1 silencing in U138MG cells, caspase-3, caspase-8, and caspase-9 were subsequently detected using Western blot analysis. HeLa cells treated with doxycycline, used to induce caspase-dependent cell death, were utilized as a positive control. Although the indicated caspases were evidently present in their inactive form, none of them was detected in the cleaved, active form in AEBP1-depleted U138MG cells, compared to the positive control. These results indicate that the caspase family does not play a role in cell death triggered by AEBP1 silencing in glioma cells. Furthermore, Bid, a mitochondrial membrane protein involved in caspase-8 activation, was also rendered inactive in AEBP1-silenced U138MG cells. Caspase-Glo 3/7 assay confirmed that caspase-3 and caspase-7 were inactive upon AEBP1 depletion, compared to the positive control. To further ascertain that cell death was not induced by the classical caspase-dependent pathway, AEBP1-depleted glioma cells were treated with Z-VAD-FMK, a pan caspase inhibitor that inhibits caspase-dependent cell death. Nevertheless, MTT cell viability assay revealed that cell death was still detected in AEBP1-depleted U138MG cells treated with the pan caspase inhibitor. On the other hand, doxycycline treated U138MG cells remained viable after the addition of the pan caspase inhibitor. These results collectively demonstrate that although caspase components are present in AEBP1-depleted U138MG glioma cells, the triggering of cell death does not depend on the classical caspase pathway of cell death. Remarkably, the pattern in which DNA is fragmented in AEBP1-depleted U138MG cells does not coincide with the nucleosomal ladder pattern generated by caspase-dependent DNA fragmentation, further affirming a caspase-independent mechanism of cell death.

Other experiments were aimed at investigating the involvement of the parthanatos pathway, a PARP-1-dependent cell death mechanism, in AEBP1-depleted U138MG glioma cells [44]. Parthanatos is characterized by the hyperactivation of poly [ADP-ribose] polymerase 1 (PARP-1) due to genomic stress, followed by PARP-1-mediated synthesis and accumulation of poly [ADP-ribose] (PAR). The accumulation of PAR causes nuclear translocation of apoptosis-inducing factor (AIF), resulting in DNA fragmentation and eventually cell death [98]. To examine if cell death in AEBP1-depleted U138MG cells occurs in a PARP-1-dependent manner, Sinha and colleagues [44] measured the levels of PARP-1 and PAR in AEBP1-depleted glioma cells. Results showed that the formation of the PARP-1 and PAR polymers increased in AEBP1-depleted cells in a time-dependent manner. PARP-1 levels increased on the 3^rd^ day after transfection of AEBP1 siRNA, while the increase in PAR polymer formation was reported on the 7^th^ day. These results strongly suggest that cell death in AEBP1-depleted U138MG glioma cells is mediated by PARP-1-dependent mechanism of cell death. It was demonstrated that AEBP1 depletion causes a loss in mitochondrial outer membrane potential and the consequent release of AIF from the mitochondria. Mitochondrial outer membrane permeabilization (MOMP) is a mitochondrial protein that triggers apoptosis. Confocal immunofluorescent analysis using MitoTracker Red CMXRos, which is used to examine MOMP integrity, revealed a time-dependent loss of MOMP in AEBP1-depleted glioma cells. Immunofluorescence and subcellular fractionation analysis revealed that AIF was released from the mitochondria and translocated to the perinuclear region on the 5^th^ day and was fully situated in the nucleus by the 9^th^ day. An increase in *γ*H2AX foci, representing double-strand breaks, was observed at the nuclear periphery and was later predominant in the core of the nucleus. These findings are in line with the mechanism by which AIF, mediated by PARP-1 and PAR, translocates to the nucleus, leading to DNA fragmentation and cell death in AEBP1-silenced U138MG cells. It is worthy of note that this study experimentally demonstrates the therapeutic advantage of targeting AEBP1 in glioblastoma, rendering the inhibition of AEBP1 as a promising therapeutic approach (Table 1). Figures 1 and 2 illustrate the main regulatory effects of AEBP1 on cancer-related proteins involved in parthanatos (PARP-1) pathway.

## 7. AEBP1 Regulates Cell Growth and Proliferation via PTEN Signaling Pathway

AEBP1 was shown to negatively regulate the activity of PTEN (phosphate and tensin homolog) [10]. Indeed, Ro and colleagues [10] demonstrated the ability of AEBP1 to suppress PTEN in adipose tissue, thereby promoting adipocyte proliferation. AEBP1 was shown to be a positive regulator of cell proliferation due to its high expression in proliferative preadipocytes, compared to terminally differentiated, nonproliferative adipocytes [11]. Besides the interplay between AEBP1 and PTEN in adipogenesis, the relationship between AEBP1 and PTEN has also been investigated in cancer. PTEN is a tumor suppressor involved in the modulation of mammalian growth and cell migration [99]. Through its lipid phosphatase activity, PTEN suppresses cell growth and motility by downregulating PI3K-Akt signaling. Thus, PTEN mutations were strongly associated with tumor development and progression [100]. Indeed, PTEN mutations occur in more than 70% of glioma cell lines, and such mutations are involved in the pathogenesis of high-grade gliomas. Interestingly, Sinha and colleagues [44] reported that cell death occurs in PTEN-proficient glioma cell line (LN18) upon AEBP1 silencing. Thus, it would prove useful to investigate whether the mechanism by which cell death occurs due to AEBP1 silencing differs between PTEN-deficient (U138MG) and PTEN-proficient (LN18) glioma cells. Intriguingly, compared to U138MG glioma cells, cell death in LN18 glioma cells was accompanied by caspase-3 activation, MOMP loss, and PARP-1 cleavage. Furthermore, upon transfection of wild-type PTEN into PTEN-deficient glioma cells (U138MG and U87MG), caspase-3 activation, cleavage of PARP-1, and nucleosomal ladder pattern of DNA fragmentation were observed, and these characteristics gradually increased on the 7^th^ and 9^th^ day after transfection. Additionally, levels of pAkt decreased upon transfection of PTEN. Collectively, these results demonstrate that cell death in PTEN-proficient glioma cells occurs in a caspase- and Akt-dependent mechanism upon AEBP1 silencing. The study also demonstrated that the presence of AEBP1 is essential for the survival of glioma cells. These findings are consistent with the role of PARP-1-dependent cell death in AEBP1-depleted glioma cells [44]. Thus, targeting AEBP1 could be a promising therapeutic intervention towards the treatment of gliomas, paving the way for more innovative and revolutionary cancer therapy (Table 1). The main regulatory effects of AEBP1 on cancer-related proteins involved in PTEN pathway are illustrated in Figures 1 and 2.

## 8. Conclusion

In this review, the role of AEBP1 in cancer development and progression, with a focus on its regulatory function in several signaling pathways implicated in carcinogenesis, was analyzed. We underscored the recent experimental evidence linking modulation of AEBP1 expression to regulation of carcinogenesis. It is evident that overexpression of AEBP1 correlates with several types of cancer, rendering AEBP1 as a potential oncogene. Due to its transcriptional repressor activity, AEBP1 was shown to regulate the expression of several proteins implicated in the development of different types of cancers. AEBP1 seems to promote tumorigenesis and regulate cancer-related events by targeting several signaling pathways including the canonical NF-*κ*B, PI3K-Akt, sonic hedgehog (Shh), p53, parthanatos (PARP-1), and PTEN. Indeed, the canonical NF-*κ*B pathway is one of the most targeted pathways by AEBP1, and the relationship between AEBP1 and the canonical NF-*κ*B pathway is crucial in many types of cancer including, but not limited to, breast cancer, skin cancer, glioblastoma, bladder cancer, gastric cancer, colorectal cancer, ovarian cancer, and leukemia. In sum, AEBP1 exerts proproliferative, antiapoptotic, prometastatic, proangiogenic, and proinflammatory effects, both *in vitro* and *in vivo*. Such effects underlie the ability of AEBP1 to promote carcinogenesis. Figure 1 summarizes the main regulatory effects of AEBP1 on cancer-related proteins and the major signaling pathways targeted by AEBP1 to promote its proproliferative, antiapoptotic, prometastatic, proangiogenic, and proinflammatory effects, triggering cancer development.  Figure 2 presents a schematic, mechanistic representation of the molecular networks and signaling pathways that underlie the molecular roles of AEBP1 in carcinogenesis. Importantly, the association of AEBP1 overexpression with tumorigenesis and carcinogenesis presents AEBP1 as a potential biomarker for cancer prognosis and a therapeutic target for the prevention and/or treatment of different types of cancer.

## Figures and Tables

**Table 1:** A summary of the *in vitro* and *in vivo* cancer-promoting effects of AEBP1.

Type of cancer	Pathway	Experimental model	Effect	Reference
Breast cancer	NF-*κ*B	Transgenic mice with AEBP1 overexpression (AEBP1^TG^)	AEBP1 overexpression promotes alveolar hyperplasia, promotes stromal macrophage infiltration, promotes proinflammatory response through NF-*κ*B/p65 upregulation, upregulates TNF*α* expression, and increases nuclear NF-*κ*B/p65 level	[28]
		Human malignant breast epithelial cells	AEBP1 is highly expressed in myoepithelium AEBP1 is associated with skeletal development AEBP1 promotes bone differentiation and ECM remodeling	[39]
	PI3K-Akt	HC11 cells	AEBP1 overexpression upregulates pAkt activity in a TNF*α*-dependent manner AEBP1 silencing downregulates pAkt level	[28]
	Sonic hedgehog (Shh)	HC11 cells	AEBP1 overexpression upregulates Gli1 and Bmi1 and promotes Shh signaling	
		HMECs & MCF-7 cells	Gli1 increases AEBP1 expression	[40, 41]
		MCF-7 cells	TSHZ2 represses AEBP1 expression	[41]
		HMECs	TSHZ2 knockdown increases AEBP1 expression	[41]

Glioblastoma	NF-*κ*B	Human GBM tumor samples	AEBP1 is highly expressed in primary GBM and it promotes cell proliferation in primary GBM	[42]
		U78MG cells	AEBP1 silencing decreases cell proliferation, downregulates expression of apoptotic regulators, inhibits cell hypoxia, upregulates MDM2, and downregulates genes that promote mitosis (Cdc20 and Cdc25C)	[7]
		U87MG and U138MG cells	AEBP1 silencing downregulates cell proliferation and upregulates apoptotic activities	[7]
		U87MG and U251MG cells	AEBP1 silencing downregulates cell proliferation, decreases cell invasion abilities, augments apoptotic effects, upregulates expression of caspase-3 and Bax, downregulates expression of NF-*κ*B1/p105, MMP-2, and Bcl-2, and decreases NF-*κ*B signaling	[43]
	PI3K-Akt	U138MG cells	AEBP1 silencing downregulates PI3KCB activity and increases expression of *γ*H2AX foci, resulting in increased double-strand breaks	[44]
		TCGA-GBM dataset	AEBP1 positively regulates PI3KCB activity	[44]
	p53	U78MG cells	AEBP1 silencing upregulates *TP53* gene	[7]
	Parthanatos (PARP-1)	U138MG cells	AEBP1 silencing increases the formation of PARP-1 and PAR polymers, promotes MOMP loss, induces AIF translocation from mitochondria to perinuclear region, and increases expression of *γ*H2AX foci predominantly in nuclear region	[44]
	PTEN	LN18 cells (PTEN-proficient)	AEBP1 silencing promotes caspase-dependent cell death and induces caspase-3 activation, MOMP loss, and PARP-1 cleavage	[44]
		U138MG and U87MG cells transfected with PTEN	AEBP1 silencing decreases pAkt levels and induces caspase-3 activation, cleavage of PARP-1, and nucleosomal ladder pattern of DNA fragmentation	[44]
Bladder cancer	NF-*κ*B	GSE13507 dataset	AEBP1 correlates with TNM staging AEBP1 decreases OS rate AEBP1 enhances myogenesis and coagulation AEBP1 promotes EMT, TNF*α* signaling, and NF-*κ*B signaling	[37]
		GSE31685 dataset	AEBP1 is associated with poor prognosis and high degree tumor staging AEBP1 promotes cell adhesion, organ development, and skeletal system	[45]
		GSE13507 and GSE120736 datasets	AEBP1 is highly expressed in NMIBC and MIBC tumors AEBP1 correlates with disease progression and poor OS rate AEBP1 stimulates activation of chemokine pathway, FOXM1 pathway, collagen formation, and ECM remodeling AEBP1 triggers ECM and EMT	[46]

Gastric cancer	NF-*κ*B	MGC803 and XN0422 cells	AEBP1 silencing suppresses cell proliferation and colony formation, inhibits cell migration, invasion, and metastasis, attenuates cell progression by suppressing EMT via inhibition of NF-*κ*B signaling, decreases NF-*κ*B/p65 and p-NF-*κ*B/p65 levels, upregulates I*κ*B*α* expression, inhibits NF-*κ*B signaling, and downregulates expression of CXCR-4 and ICAM-1	[47]
		Subcutaneous xenograft model in nude mice	AEBP1 expression correlates with increased tumor weight and size AEBP1 silencing decreases NF-*κ*B/p65 and p-NF-*κ*B/p65 levels and attenuates cell progression by suppressing EMT via inhibition of NF-*κ*B signaling	[47]

Colorectal cancer	NF-*κ*B	Human CRC tissue	AEBP1 is highly expressed in nucleus and cytoplasm AEBP1 correlates with lymph node metastasis and TNM staging. AEBP1 correlates with poor OS and DFR rate	[48]
		HT-29 cell	AEBP1 silencing suppresses cell proliferation and upregulates apoptotic activities	[48]
		Human COAD tissue	AEBP1 correlates with advanced clinicopathological features and poor OS AEBP1 increases expression of MMP-2, vimentin, TWIST, ZEB1, ZEB2, and SNAIL1 AEBP1 correlates with increased tumor size, lymph node metastasis, and tumor staging	[49]
		DLD-1 and LoVo cells	AEBP1 silencing decreases cell proliferation and colony formation, suppresses cell migration and invasion, suppresses expression of MMP-2, vimentin, and TWIST, suppresses NF-*κ*B/p65 and p-NF-*κ*B/p65 levels, and increases E-cadherin expression	[49]
		SW1116 cells	AEBP1 overexpression augments cell proliferation, migration, and invasion, increases expression of MMP-2, vimentin, and TWIST, enhances NF-*κ*B/p65 and p-NF-*κ*B/p65 levels, and decreases E-cadherin expression AEBP1 mediates expression of EMT-related components	[49]
		Athymic BALB/c nude mice	AEBP1 silencing decreases tumor weight and volume, inhibits rate of cell proliferation, and reduces number of metastatic lung nodules	[49]
Ovarian Cancer	NF-*κ*B	TCGA, GSE26712, and GSE51088 datasets	AEBP1 is highly expressed in SOC AEBP1 correlates with poor OS rate AEBP1 localizes in the ECM AEBP1 regulates cell adhesion and collagen remodeling	[50]
		OVCAR3	AEBP1 expression is promoted by TGF*β*1	[50]
		TCGA, GSE26193, and GSE9891 datasets	AEBP1 is associated with progression of tumor stage (stages I–IV) AEBP1 demonstrates strong interaction with MMP-2 and ECM components	[21]
		GSE49997, GSE17260, TCGA.RNASeqV2, and PMID158975 datasets	AEBP1 is associated with progression of tumor stage (stages I–IV)	[21]

Skin cancer	NF-*κ*B	Human BCC, SCC, and MM stromal fibroblasts	AEBP1 correlates with CAFs in MM tumors	[51]
		Nude mice injected with Mel-CVR cells	AEBP1 expression is upregulated in the indicated cells AEBP1 knockdown decreases cell viability AEBP1 decreases I*κ*B*α* expression AEBP1 upregulates NF-*κ*B/p65 activity	[52]
			AEBP1 knockdown decreases tumor size	
		Human BCC tissue	AEBP1 expression is upregulated in telogen phase	[53]
	PI3K-Akt	Mel-CVR18 and Mel-CVR21 cells	AEBP1 transcriptional activity is augmented by CREB-BR AEBP1 expression and cell viability are suppressed by CREB knockdown AEBP1 expression decreases upon inhibition of PI3K-Akt signaling by LY294002, a PI3K-Akt inhibitor AEBP1 overexpression is mediated by PI3K-Akt-CREB pathway	[52]

Childhood acute lymphoblastic leukemia (cALL)	PI3K-Akt	GSE28460 and GSE60926 datasets	AEBP1 is highly expressed in the relapse stage AEBP1 overexpression upregulates PI3K-Akt-enriched genes (TCL1A, THBS1, TLR4, PDGFC, CSF3R, ITGB3, IL6R, ITGA2B, and VEGFA)	[54]
		GSE67684 dataset	AEBP1 expression is downregulated in the posttreatment stage	[54]
	p53	Jurkat, Nalm-6, and Raji cells	AEBP1 expression is upregulated in these cancer cell lines	[54]
		Jurkat cells	AEBP1 silencing inhibits cell proliferation rate, suppresses Bcl-2 expression, decreases percentage of cells in S phase, and increases percentage of cells in G1 phase AEBP1 increases expression of caspase-3, caspase-7, and Bax, inducing apoptosis AEBP1 mediates cell survival and blocks apoptosis	[54]
